# Montmorency cherry supplementation enhances 15 km cycling time trial performance: Optimal timing 90‐min pre‐exercise

**DOI:** 10.1002/ejsc.12187

**Published:** 2024-08-30

**Authors:** Jimmy T. Wangdi, Mary F. O’Leary, Vincent G. Kelly, Jonathan C. Y. Tang, Joanna L. Bowtell

**Affiliations:** ^1^ BioActivEx, Public Health and Sport Sciences St Luke's Campus University of Exeter Medical School Devon UK; ^2^ School of Human Movement and Nutrition Sciences University of Queensland Brisbane Queensland Australia; ^3^ School of Exercise and Nutrition Sciences Queensland University of Technology Brisbane Queensland Australia; ^4^ Bioanalytical Facility Norwich Medical School University of East Anglia Norwich UK; ^5^ Clinical Biochemistry Departments of Laboratory Medicine Norfolk and Norwich University Hospital NHS Foundation Trust Norwich UK

**Keywords:** endurance exercise, Montmorency cherry, nitric oxide, time trial, training status

## Abstract

Montmorency cherry (MC) can improve endurance performance, but optimal pre‐exercise timing of supplementation and influence of training status on efficacy are unknown. We investigated the effect of MC concentrate ingestion between 30‐ and 150‐min pre‐exercise in trained and recreational cyclists on 15‐km time trial (TT) performance and exercise economy. Twenty participants (10 recreationally active, RA; 10 trained, T) completed 10 min of steady‐state exercise (SSE) at 40%Δ (SSE) and a TT on four separate occasions following an unsupplemented (US), 30‐, 90‐ or 150‐min pre‐exercise Montmorency cherry concentrate (MCC) supplementation conditions (MCC^30/90/150min^). Venous and capillary blood samples were taken at regular intervals pre‐ and post‐SSE and TT. MCC significantly improved TT performance, but not exercise economy. The greatest improvement in performance occurred following MCC^90min^ compared to US (US 1603.1 ± 248 s vs. MCC^90min^ 1554.8 ± 226.7 s, 2.83% performance improvement). Performance was significantly enhanced for trained (US 1496.6 ± 173.1 s vs. MCC^90min^ 1466.8 ± 157.6 s) but not recreationally active participants. Capillary [lactate] and heart rate were significantly greater during the TT for the 90‐min dose timing (*p* < 0.05). In the MCC^30min^ and MCC^90min^ conditions, plasma ferulic (US 8.71 ± 3.22 nmol. L^−1^ vs. MCC^30min^ 15.80 ± 8.69 nmol. L^−1^, MCC^90min^ 12.65 ± 4.84 nmol. L^−1^) and vanillic acid (US 25.14 ± 10.91 nmol.L^−1^ vs. MCC^30min^ 153.07 ± 85.91 nmol. L^−1^, MCC^90min^ 164.58 ± 59.06 nmol. L^−1^) were significantly higher pre‐exercise than in US and MCC^150min^ conditions (*p* < 0.05). There was no significant change in muscle oxygenation status or plasma nitrite/nitrate concentration. MCC supplementation enhanced endurance exercise performance optimally when consumed ∼90 min pre‐exercise producing maximal plasma phenolic metabolites during exercise. The ergogenic effect was greater for trained participants.

## Highlights

- Acute single‐dose supplementation of Montmorency cherry concentrate (MCC) (>800 mg polyphenols) improved endurance cycling time trial (TT) performance by 2.83% or 48.3 s.
- Supplementation 90 min prior to exercise produced the greatest increases in plasma phenolic metabolites and improvement in performance, suggesting the ergogenic effects are mediated by these metabolites.
- Trained but not recreationally active (RA) participants experienced ergogenic effects from cherry supplementation throughout the TT.

## 1 INTRODUCTION

Montmorency cherry (MC) supplementation can improve endurance and sprint exercise performance (Keane et al., 2018; Levers et al., 2016; Morgan et al., 2019). Acute (Keane et al., 2018) and chronic supplementation, with the last dose consumed 60 min prior to exercise (Morgan et al., 2019), have proved effective; however, the optimal strategy for the timing of supplement intake prior to endurance exercise is unclear. Timing is likely to influence supplement effectiveness, as the hypothesised underpinning mechanisms rely on the bioavailability and antioxidant effects of phenolic metabolites, which peak in plasma ∼1–‐2 h post‐ingestion (Keane, Bell, et al., 2016).

Six studies have investigated the effects of MC supplementation on exercise performance (Clifford et al., 2013; Davis et al., 2019; Howatson et al., 2010; Keane et al., 2018; Levers et al., 2016; Morgan et al., 2019). Five of these studies (Clifford et al., 2013; Davis et al., 2019; Howatson et al., 2010; Levers et al., 2016; Morgan et al., 2019) utilised a chronic supplementation strategy, with a final dose consumed 60–90 min prior to exercise. Only Keane et al. (2018) provided a single acute dose, showing a significant improvement in end sprint exercise performance (20 s peak power (∼9%) and 60 s all‐out sprint (∼10%) on a cycle ergometer) following MC consumption 90‐min pre‐exercise (Keane et al., 2018). However, these ergogenic effects were observed during sprint exercise performed following severe intensity exercise. No significant effects of MC supplementation were observed for time to exhaustion during this bout of severe intensity exercise.

Only 2 (Levers et al., 2016; Morgan et al., 2019) of 5 studies examining the effects of chronic MCC supplementation on endurance performance showed a significant improvement compared to the control group. The absence of MCC effects in these studies can likely be explained by the final dose timing (2–‐3 h pre‐exercise (Clifford et al., 2013) and the low polyphenol dose (200 mg Davis et al., 2019). Optimal polyphenol dose for enhanced muscle recovery and exercise performance appears to be between 600 and 1200 mg (Sabou et al., 2021), although different analytical methods make comparisons of phenolic content between studies difficult (Sabou et al., 2021). Studies assessing the effects of acute MC supplementation on endurance exercise performance, optimal timing of supplementation and mechanisms of action are needed to resolve the conflicting findings.

Four (Clifford et al., 2013; Keane et al., 2018; Levers et al., 2016; Morgan et al., 2019) of the 6 studies investigating the performance effects of MC supplementation recruited trained individuals. Currently, MC supplementation has only been observed to enhance performance for trained participants, but a direct comparison of MC efficacy between trained and RA participants has not yet been conducted. It is plausible that efficacy is influenced by training status as observed with another polyphenol blend (New Zealand blackcurrant (NZB)), where repeated sprint performance was enhanced only for trained individuals (Godwin et al., 2017).

The ergogenic effects of acute MC supplementation are likely mediated by elevated plasma phenolic metabolites and their biological effects. These effects are thought to be (1) improvements in muscle perfusion (Cook et al., 2017; Trexler et al., 2014) via increased NO bioavailability due to reduced ROS production and subsequent degradation of NO to peroxynitrite; (2) inhibition of ROS producing enzymes like NADPH oxidase (Rodriguez‐Mateos et al., 2013) and subsequently reduced redox perturbations impacting redox sensitive structures like sarcoplasmic reticulum during exercise; and (3) anti‐inflammatory effects mediated via the downregulation of the cyclooxygenase (COX) pathway (Seeram et al., 2001) leading to ergogenic analgesic effects similar to those observed with NSAIDs (Holgado et al., 2018; Mauger et al., 2010; Seeram et al., 2001). Therefore, to maximise these effects, it is important to time the peak of plasma phenolic metabolite concentration with exercise. Previous research has shown phenolic metabolites peak in the plasma ∼1–‐2 h post‐ingestion (Keane, Bell, et al., 2016). Thus, ensuring consumption of MC occurs in this timeframe relative to exercise onset was hypothesised to produce a greater improvement in exercise performance than supplementing prior to or after this period.

This study assessed the effects of acute MC concentrate (MCC) supplementation on 15‐km cycling TT performance and sub‐maximal exercise economy and whether supplementation timing (30–150 min) and training status influenced the observed effects.

## 2 MATERIAL AND METHODS

### 2.1 Participants

Twenty participants (13 male, 7 female, aged 29 ± 6 years, height 174.6 ± 8.6 cm, weight 69.91 ± 9.66 kg, *V̇*O_2max_ 3.60 ± 0.97 L min^−1^/51.47 ± 11.48 mL kg^−1^ min^−1^ and relative peak power output (PPO) 4.64 ± 0.96 W kg^−1^) asymptomatic of illness and injury were recruited to take part. Sample size calculations were based on the expected difference between MCC and placebo (PLA) on exercise performance (effect size of 1), plasma phenolic metabolite concentration data from (Keane, Bell, et al., 2016) and 15‐km cycling TT performance data from Morgan et al. (2019). Exclusion criteria included injury or serious illness six months prior, current use of long‐term medication including anti‐inflammatory drugs or sleep medication, fruit allergies and overweight/obese individuals (BMI of ≥25 kg m^−2^). Participants were screened for underlying illness and injury and assessed for readiness to exercise (PARQ) prior to giving written and informed consent before testing.

Participants were categorised into two training status groups, endurance‐trained and RA, determined by relative *V̇*O_2max_ (mL kg^−1^ min^−1^) and relative PPO (W kg^−1^) and obtained from cardiopulmonary exercise testing (CPET) during the familiarisation visit. Groups were based on performance levels described by (Pauw et al., 2013; Decroix et al., 2015) (Table 1). A pooled performance level was calculated from the relative *V̇*O_2max_ performance level and relative PPO performance level (Equation (1)). A pooled performance level of ≥3 was categorised as endurance trained; a pooled level of <3 was categorised as RA. Categorisation values are available in the supplementary material (SDC Table S1).

**TABLE 1:** Mean performance categorisation values by the group.

Performance level	Mean *V̇*O_2max_ (mL kg^−1^ min^−1^)			Peak power output (W kg^−1^)			*N*		
	Total	Males	Females	Total	Males	Females	Total	Males	Females
Recreationally active	44.19 ± 7.64	47.52 ± 5.38	36.40 ± 6.79	3.91 ± 0.69	4.13 ± 0.66	3.40 ± 0.52	10	7	3
Trained	58.75 ± 10.10	65.70 ± 5.74	48.32 ± 3.08	5.37 ± 0.54	5.69 ± 0.38	4.89 ± 0.35	10	6	4

**Equation 1:** Pooled Performance Level

(1) $$\text{Pooled}\;\text{Performance}\;\text{Level}=\frac{\left({VO}_{2\;\max}\;\text{Performance}\;\text{Level}+\text{PPO}\;\text{Performance}\;\text{Level}\right)}{2}$$

This study had ethical approval from the [Human Research Ethics Committee at the University of Queensland] and reciprocal approval from the [Sport and Health Sciences Ethics Committee at the University of Exeter].

### 2.2 Experimental design

Experimental trials required participants to complete four laboratory visits. Each visit consisted of 10 min of steady‐state exercise (SSE) at 40%Δ (SSE) on a cycle ergometer (Lode Corival CPET, Lode BV, Groningen, The Netherlands), followed by a 5‐min rest before a 15‐km TT on a cycle ergometer (Watt Bike Trainer, West Bridgford, United Kingdom). Tests occurred following an unsupplemented (US) condition and an acute undiluted 60 mL dose of commercially available MCC (ActiveEdge Ltd, Hanworth, Middlesex, United Kingdom) 30 min, 90 min or 150 min prior to exercise (MCC^30/90/150min^). MCC was refrigerated prior to consumption. Following consumption, participants were instructed to swill out the cup with water to ensure all juice had been consumed. The analysis of MCC phenolic content (Chandra et al., 2001) was conducted by Atlas Bioscience, Inc. (Tucson, AZ; anthocyanin analysis by high performance liquid chromatography, total polyphenols by the Folin–Ciocâlteu method). Total content was 834 mg polyphenolics, 2556 mg total anthocyanins, with pelargonidin (1362 mg) and delphinidin (678 mg) the most prevalent anthocyanins. Full details of supplement analysis are available in the supplementary material (SDC Table S2).

Test order was randomised by Latin square counterbalance. Each test was separated by a washout period of ≥7 days. Testing sessions for female participants were completed in the luteal menstrual phase (determined by questionnaire adapted from the Women's Health Symptom Survey Questionnaire) or ∼30 days apart if phases were unidentifiable (e.g., due to contraception). This was to mitigate potential effects of the menstrual cycle phase on exercise performance.

### 2.3 Familiarisation visit

Participants performed a 5‐min warm‐up at a self‐selected cadence and power output, which was repeated for each subsequent visit. Participants then completed a CPET to determine power output at the gaseous exchange threshold (PO^GET^) and *V̇*O_2max_ (PPO). Participants were then familiarised with the TT protocol and equipment.

PPO was assessed using an incremental ramp exercise test on a cycle ergometer (Lode Corival cpet, Lode BV, Groningen, The Netherlands), commencing at 50 W for 3 min, before increasing workload at 20 W min^−1^. Participants pedaled at a continuous pre‐selected cadence and until volitional exhaustion or a drop ≥10 rpm from the pre‐selected cadence. *V̇*O_2_ was assessed by breath‐by‐breath indirect calorimetry (Metamax 3b, Cortex), and heart rate (HR) was measured continuously throughout (Polar Electro). The indirect calorimeter was calibrated for gas and volume prior to each test, using a reference gas (16.35% O_2_ and 4.28% CO_2_) and a 3‐L volume calibration syringe (Hans Rudolf Inc. Kansas City, MO, USA). *V̇*O_2max_ was defined as the highest 30‐s rolling average across each test. The Metamax 3b has previously been shown to be reliable within <2% for measurement of VE, *V̇*O_2_ and *V̇*CO_2_ (Macfarlane et al., 2012).

Values for absolute PO^GET^ and absolute PPO determined during the CPET were used to calculate SSE intensity at 40%Δ, using Equation (2).

**Equation (2)**: Calculation of 40%Δ Steady State Exercise Intensity

(2) $$\text{SSE}\;\text{intensity}\;\text{at}\;40\%\Delta =\left(\left(\text{PPO}\;-\;{\text{PO}}^{\text{GET}}\right)\times 40\%∆\right)+{\text{PO}}^{\text{GET}}$$

40%Δ was used as it has been shown to be of a similar intensity to 70% *V̇*O_2max_ (Lansley et al., 2011), whilst producing less variation in intensity between participants. An equivalent intensity to 70% *V̇*O_2max_ was used in line with similar previous studies (Deley et al., 2017; Oh et al., 2010).

### 2.4 Pre‐testing protocol

Participants were instructed to avoid increased consumption of foods with high polyphenol concentrations pre‐supplementation, in addition to avoiding alcohol and caffeine consumption 48 h before each visit. Participants also kept detailed food and beverage diaries in the 48 h prior to their first exercise test, which were replicated as closely as possible prior to each subsequent test. Participants were instructed to maintain habitual physical activity levels throughout the trial period, repeating any activity during the 48‐h period prior to the first visit for subsequent visits.

### 2.5 Experimental protocol

Participants were provided with a standardised breakfast (1.5‐h pre‐exercise) before all exercise testing to help maintain ecological validity. The standardised breakfast was pre‐packaged flavored oats (Uncle Tobys Foods Pty Ltd), and participants were given a choice to consume one or two 46 g sachets (30.5 g carbohydrate), with this portion size being maintained for each test. Participants were instructed to make the breakfast with their choice of milk/milk substitute or water, maintaining this choice for each test. Complete nutritional information for one sachet is available in the supplementary material (SDC Table S3).

Venous blood samples were taken before and after exercise during each visit and prior to supplementation during supplemented visits. Baseline blood samples occurred pre‐supplementation. Pre‐exercise blood draws occurred approximately 15 min prior to the start of SSE and post‐exercise within 5 min of exercise cessation. SSE tests were performed over 10 min at 40%Δ (as in Casaburi et al., 1987), during which gaseous exchange data was measured using breath‐by‐breath indirect calorimetry. During SSE tests and TTs, muscle oxygenation was indirectly assessed by near‐infrared spectroscopy (NIRS) (PortaMon, Artinis Medical Systems B.V. Einsteinweg 17, 6662 PW Elst). The NIRS device was positioned on the vastus lateralis at the midpoint between the iliac crest and the distal femur lateral epicondyle. Recorded measures included mean tissue saturation index (TSI), combined oxy‐haemoglobin and myoglobin ([O_2_(Hb + Mb)]), combined deoxy‐haemoglobin and myoglobin ([H(Hb + Mb)]) and combined total hemoglobin and myoglobin ([t(Hb + Mb)]). TSI ½ recovery time (TSI ½ RT) was calculated as the time taken to reach ½ of baseline TSI% over 30 s following SSE and TT. The baseline was defined as the period of 20 s with the highest mean value during the 5‐min rest period following the warmup and pre‐exercise onset.

**FIGURE 1:**
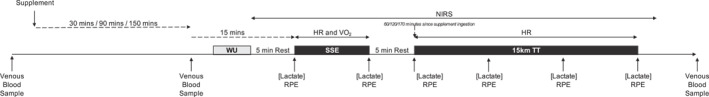
Experimental protocol showing timeline from baseline to final venous blood sample. Duration between supplement ingestion and exercise onset varied. Protocol start point is also displayed for unsupplemented (US) condition. Warm up was performed over 5 min, steady‐state exercise was performed over 10 min, and 15‐km time trial duration varied with performance between participants. Points of data collection for physiological measures are displayed (Capillary blood lactate concentration ([Lactate]), heart rate (HR), rate of perceived exertion (RPE), oxygen uptake (V̇*O*_*2*_) and near infrared spectroscopy (NIRS)).

During all tests, HR, the rate of perceived exhaustion (RPE) (Borg scale) and fingertip capillary blood lactate concentration ([lactate]) (LactatEDGE, Warszawa, Poland) were measured. RPE and [lactate] were measured pre‐exercise and post‐exercise for SEE and TT testing and every 5 kms completed during TTs. The LactatEDGE analyser has a coefficient of variation of 4.0% and a within‐subject standard deviation of ±0.35 (Bonaventura et al., 2015). The full experimental protocol is displayed in Figure 1.

### 2.6 Blood analysis

Venous blood samples were collected in vacutainer tubes containing lithium heparin. Samples were centrifuged at 4500 rpm for 15 min at 4°C. Plasma samples were stored at −80°C until analysis.

### 2.7 Plasma TNF*α* analysis

Plasma samples were analyzed for the content of TNF*α* ([TNF*α*]) by milliplex multi‐analyte profiling human high‐sensitivity T‐cell magnetic bead panel assay (Merck Millipore, Burlington, Massachusetts, USA) according to the manufacturer's instructions. The inter‐assay precision of <15%, intra‐assay precision of <10% and assay accuracy of 98%–107% are reported by the manufacturer.

### 2.8 Plasma nitrate and nitrite analysis

Plasma concentrations of NO_3_^−^ ([NO_3_^−^]) and [NO_2_^−^] were assessed by ozone‐based chemiluminescence (Sievers NO analyser, model 280i, GE Water and Process Technologies Analytical Instruments) as described previously (Wylie et al., 2013). Analysis liquid Program (Version 3.21, GE Water and Process Technologies Analytical Instruments) was used to integrate chemiluminescence signal peaks in mV and determine the area under the curve of injections for nitrate. OriginLab (Version 9.7 SR1, OriginLab Corporation) was used to manually integrate chemiluminescence signal peaks in mV and determine the area under the curve of injections for nitrite. Nitrite data was smoothed in OriginLab by loess smoothing, with a span of 0.01.

### 2.9 Plasma phenolic metabolites profile measurement by liquid chromatography tandem mass spectrometry (LC‐MS/MS)

Phenolic metabolite analyses were performed at the Bioanalytical Facility, University of East Anglia. Plasma concentrations of protocatechuic acid, 4‐hydroxybenzoic acid, hippuric acid, vanillic acid, ferulic acid and isoferulic acid were quantified using a Waters Xevo TQ‐XS tandem mass spectrometer coupled with an Acquity I‐class ultra high‐pressure liquid chromatography pump (UPLC) system (Waters Corp.). The LC‐MS/MS system was operated in a negative electrospray ionisation (ESI ‐ve) mode. Identification and quantification were based on multiple reaction monitoring of the respective compound‐specific precursor to product ion mass to charge (m/z) transitions: protocatechuic acid 152.9 > 109.2, 4‐hydroxybenzoic acid 137.0 > 92.9, hippuric acid 178.0 > 134, vanillic acid 167.0 > 151.9, ferulic acid and isoferulic acid 193.0 > 177.9. Chromatographic separation was achieved using a ModusCore C18 reverse phase column (2.1 m × 50 mm, 2.7 μm) (Chromatography direct) maintained at a temperature of 40°C. Mobile phase A consisted of 1% acetic acid in LCMS grade deionised water with LCMS grade methanol as mobile phase B. The binary gradient program was 0 min 1% B, 0–1 min 1% B with a linear increase to 45% B at 10 min, 10–10.5 min 95% B and held to 12 min and returned to 1% B at 12.5 min to re‐equilibrate with a cycle time of 15 min. The mobile phase flow rate was 0.5 mL per min throughout the run.

The plasma sample extraction procedure was described in (Wangdi et al., 2022). In brief, 200 μL of plasma, calibration standards (blank plasma spiked with European Pharmacopoeia (EP) reference standards obtained from Merck Germany) and plasma quality controls were spiked with 20 μL of internal standard containing ferulic acid‐[^2^H_3_] (100 nmol L^−1^) and hippuric acid [^13^C_6_] (200 μmol/L) (Toronto Research Chemicals) in 0.1% formic acid (Merck) into microcentifuge tubes and mixed. To this, 1 mL of methanol was added slowly with gentle mixing and the mixture was then incubated at room temperature for 15 min, followed by centrifugation at 14,000 rpm for 7 min. The supernatant was transferred to a borosilicate glass tube and placed in an evaporator to dry under a constant stream of nitrogen at a temperature of 60°C. To the dried supernatant, 200 μL of methanol (Merck) with 0.1% formic acid was added into each tube and vortex‐mixed for 30 s, followed by 2.5 mL of ethylacetate (Merck) and vigorously mixed for 10 min. After centrifugation at 4000 rpm for 10 min, 2 mL of the ethylacetate in the upper layer was transferred to a fresh set of borosilicate glass tubes and again evaporated to dryness as described above. The dried residue was resuspended in 250 μL of LCMS grade deionised water with 1% acetic acid (Merck), then vortex‐mixed followed by centrifugation at 4000 rpm for 10 min. The final mixture was transferred into polypropylene autosampler vials, and 50 μL was injected into the LC‐MS/MS for analysis. MassLynx version 4.2 and QuanLynx software (Waters Corp.) were used for system control, data acquisition, baseline integration and peak quantification. Assay performance is summarised in SDC Table S4.

### 2.10 Data analysis

Mean values for NIRS and *V̇*O_2_ were taken during the final 5 min of SSE to remove influences of kinetic responses. Data were analyzed separately for SSE and TT using repeated measures ANOVA with a between subjects' factor of training status and Bonferroni Post‐hoc tests. Post‐hoc tests compared experimental trials to US. Data were checked for sphericity using Mauchly's test. Non‐spherical results were Bonferroni adjusted. Where outliers of greater than the mean ± 2SD were removed, values were replaced with z‐scores. For clarity, only significant main and post‐hoc effects are presented in tables and figures. Statistical analysis was performed using IBM SPSS statistics (Version 25). The alpha level of significance was set at *p* ≤ 0.05.

## 3 RESULTS

### 3.1 TT performance

**FIGURE 2:**
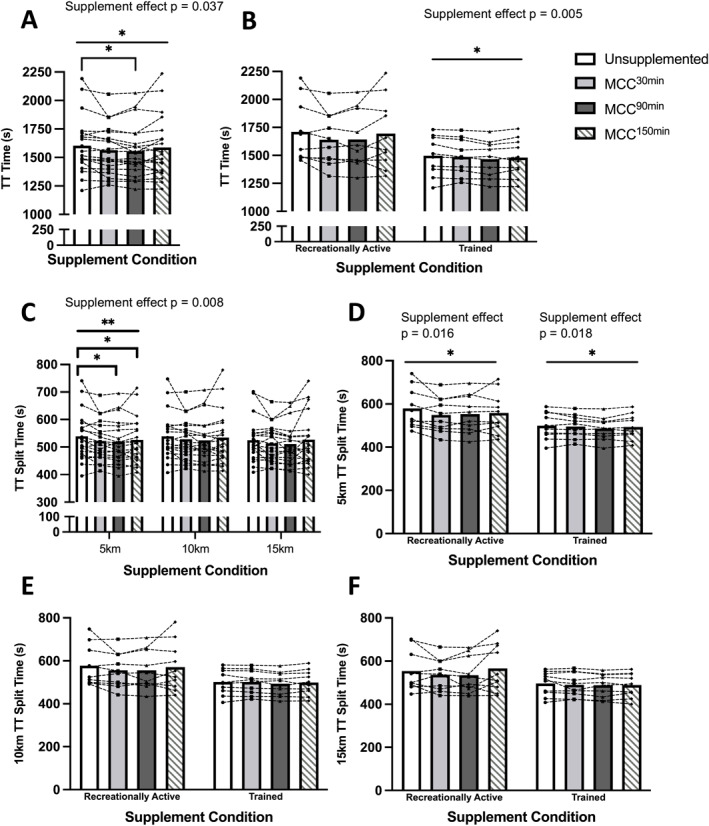
Comparison of time trial (TT) performance time for unsupplemented control and Montmorency cherry concentrate (MCC) supplementation 30 min, 90 and 150 min prior to exercise for (A) total TT time, (B) total TT time by training status, (C) TT time by 5 km split, (D) TT time for 5‐km split by training status, (E) TT time for 10‐km split by training status (F) TT time for 15‐km split by training status. Values are displayed as means and individual data points. *denotes a significant effect of supplementation timing (*p < 0.05/**p < 0.01). Bars represent supplementation timing main effects, and brackets indicate where a post‐hoc difference has been identified between two or more supplementation timings. *N* = 20.

MCC supplementation significantly improved TT time to completion (TT^Time^) (supplement effect *p* = 0.037, Figure 2A). Post‐hoc analysis revealed a significant improvement in TT^Time^ for MCC^90min^ versus US (US 1603.1 ± 248.0 s vs. MCC^90min^ 1554.8 ± 226.7 s, *p* = 0.034) but not MCC^30min^ or MCC^150min^. Analysis by training status showed a significant supplement effect on TT^Time^ for trained but not RA participants (Figure 2B).

The analysis of TT split times showed significantly improved performance at the 5‐km TT split (TT^5km^) (supplement effect *p* = 0.008), with post‐hoc tests showing significantly faster split times at TT^5km^ for MCC^90min^ and MCC^150min^ (US 539.0 ± 86.8 s vs. MCC^90min^ 519.5 ± 78.3 s vs. MCC^150min^ 525.7 ± 83.2 s, *p* = 0.020 and *p* = 0.014, respectively, Figure 2C). This effect of pre‐exercise supplementation timing on TT^Time^ was present for both training status groups at TT^5km^ (Figure 2D) but not at TT^10km^ or TT^15km^ (Figure 2E,F).

There was no significant effect of trial order on TT^Time^ for all subjects (*p* = 0.818) or for trained (*p* = 0.388) or RA participants (*p* = 0.186).

### 3.2 Capillary blood [lactate], heart rate and RPE during SSE and 15 km TT

During SSE, there was no significant effect of supplementation conditions on cycling economy, average HR (SSHR^Av^), end‐exercise RPE or change in capillary blood [lactate] (SS[Lactate]Δ) during SSE for all participants or for either training status group (SDC Table S5). There was no significant effect of supplement conditions on end SSE [lactate] for all participants (US: 9.7 ± 4.1 mM, MCC^30min^ 10.0 ± 2.6 mM: MCC^90min^ 10.6 ± 4.1 mM, MCC^150min^: 9.1 ± 3.4 mM, *p* = 0.179) or for either training status group (trained *p* = 0.211, RA *p* = 0.571).

HR, [lactate] and RPE data from TTs are presented in Table 2. TT^5km^ split data are presented in SDC Table S6. HR was significantly higher with supplementation at TT^5km^ (*p* = 0.009) and TT^10km^ (*p* = 0.011) splits. Post‐hoc tests showed a significantly increased HR at both splits during MCC^90min^ versus US condition (TT^5km^ *p* = 0.039, TT^10km^ *p* = 0.035). Analysis by training status showed a significant supplement effect on HR for the trained group at TT^5km^ (*p* = 0.036) but not the RA group.

**TABLE 2:** Physiological and perceived exertion metrics collected during time trial exercise for an unsupplemented condition and Montmorency cherry concentration supplementation 30, 90 and 150 min prior to exercise.

		US			MCC30 min			MCC90 min			MCC150 min			Supplement Effect (*p*)		
		RA	Trained	Total	RA	Trained	Total	RA	Trained	Total	RA	Trained	Total	RA	Trained	Total
Total TT	Average HR (BPM)	162 ± 18	171 ± 5	166 ± 14	162 ± 15	172 ± 5	167 ± 12	164 ± 15	170 ± 12	167 ± 14	164 ± 13	174 ± 6	169 ± 11	0.559	0.539	0.522
Pre‐TT	HR (BPM)	106 ± 15	106 ± 15	106 ± 15	105 ± 15	108 ± 12	107 ± 13	106 ± 13	108 ± 13	107 ± 12	107 ± 20	105 ± 10	106 ± 16	0.734	0.796	0.738
	Capillary [lactate] (mmol L^−1^)	7.0 ± 2.9	7.4 ± 3.9	7.2 ± 3.3	7.6 ± 2.6	7.4 ± 3.6	7.5 ± 3.1	7.8 ± 3.1	7.2 ± 4.3	7.5 ± 3.7	7.6 ± 3.3	6.2 ± 2.9	6.9 ± 3.1	0.899	0.414	0.800
	Borg RPE	10.0 ± 1.8	9.4 ± 2.0	9.7 ± 1.8	9.7 ± 2.5	9.6 ± 2.3	9.7 ± 2.3	9.2 ± 2.2	9.6 ± 1.7	9.4 ± 1.9	10.0 ± 1.3	9.1 ± 2.0	9.6 ± 1.7	0.321	0.641	0.807
Post‐TT	HR (BPM)	180 ± 14	184 ± 8	182 ± 11	183 ± 14	186 ± 6	184 ± 10	181 ± 19	186 ± 7	184 ± 14	182 ± 15	186 ± 7	184 ± 12	0.485	0.607	0.822
	Capillary [lactate] (mmol L^−1^)	11.3 ± 2.6	13.6 ± 3.0	12.4 ± 3.0	12.6 ± 3.9	14.8 ± 2.5	13.7 ± 3.4	14.5 ± 3.3	13.9 ± 4.1	14.2 ± 3.6	13.1 ± 4.0	14.1 ± 2.5	13.6 ± 3.3	0.182	0.625	0.323
	Borg RPE	17.9 ± 2.6	18.9 ± 1.0	18.4 ± 2.0	17.2 ± 2.7	19.7 ± 0.5	18.5 ± 2.3	17.5 ± 2.7	19.5 ± 0.7	18.5 ± 2.2	17.4 ± 2.7	19.7 ± 0.7	18.6 ± 2.2	0.337	0.012*	0.925

*Note*: Data is displayed for recreationally active participants (*N* = 10), trained participants (*N* = 10) and all participants (total, *N* = 20) for the total time trial and pre‐ and post‐TT. The main effect of the supplement condition is displayed for all three groups.

Abbreviations: BPM, beats per minute; MCC^30mins^/MCC^90mins^/MCC^150mins^, Montmorency cherry concentration supplementation 30, 90 and 150 prior to exercise; RA, recreationally active; RPE, rate of perceived exertion.

Blood [lactate] was significantly higher with supplementation at TT^10km^ (*p* = 0.001) but not TT^5km^ or TT^15km^. Post‐hoc tests showed a significantly higher [lactate] at TT^10km^ for MCC^30min^ compared to US (*p* = 0.007). [Lactate] was significantly higher after MCC supplementation in the RA group at TT^10km^ (*p* = 0.002). In this RA cohort, post‐hoc tests showed a significantly greater [lactate] for MCC^90min^ compared to US (*p* = 0.002). Following MCC supplementation post‐TT RPE was significantly higher in trained participants.

### 3.3 Skeletal muscle oxygenation during SSE and 15 km TT

**FIGURE 3:**
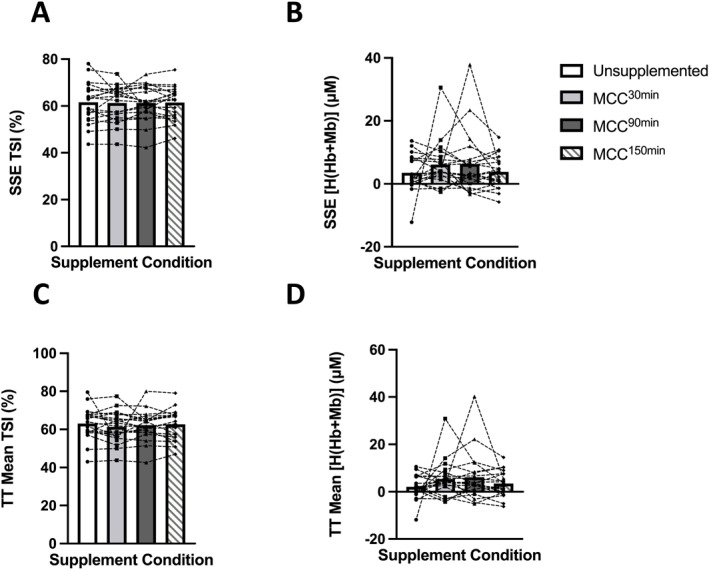
Effect of unsupplemented control and Montmorency cherry concentrate (MCC) supplementation 30 min, 90 and 150 min prior to exercise on (A) mean tissue saturation index (TSI) during steady‐state exercise (SSE), (B) mean combined deoxyhaemoglobin and myoglobin [H(Hb + Mb)] during SSE, (C) mean TSI during time trial (TT) TT, (D) mean [H(Hb + Mb)] during TT. Values are displayed as means and individual data points. *N* = 20.

There was no significant difference between supplementation trials for pre‐exercise TSI (*p* = 0.241), [O_2_(Hb + Mb)] (*p* = 0.096), [H(Hb + Mb)] (*p* = 0.292) or [t(Hb + Mb)] (*p* = 0.080) (SDC Figure S1). There were no significant differences in measures of muscle oxygenation during SSE or TT (TSI and [H(Hb + Mb)] shown in Figure 3, [O_2_(Hb + Mb)] and [t(Hb + Mb)] available in SDC Figure S2) whether for the whole cohort or for trained and RA groups. There was no significant difference between conditions in TSI ½ RT for either SSE or TT (SDC Figure S3).

### 3.4 Plasma TNF*α* concentrations

**FIGURE 4:**
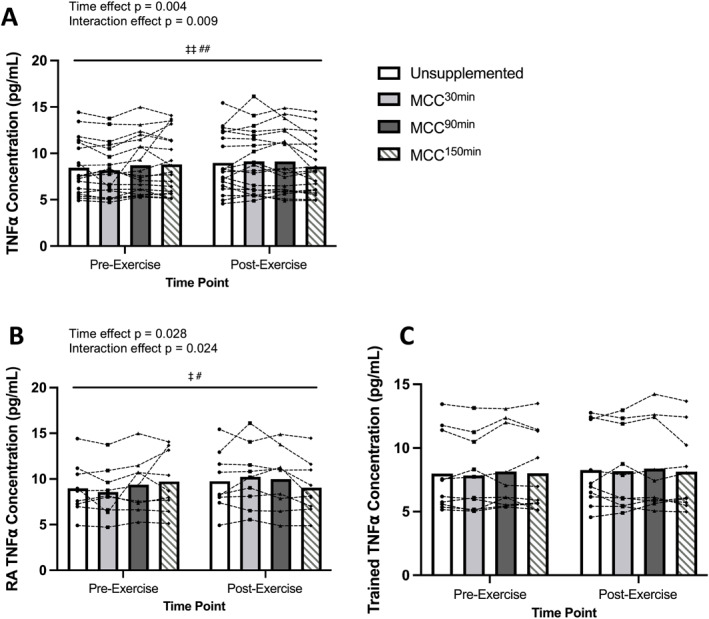
Comparison of unsupplemented control and Montmorency cherry concentrate (MCC) supplementation 30 min, 90 and 150 min prior to exercise for (A) Tumor Necrosis Factor alpha (TNF*α*) concentration for all participants (*N* = 20), (B) TNF*α* concentration for recreationally active participants (*N* = 10), (C) TNF*α* concentration for trained participants (*N* = 10), pre‐supplement, post‐supplement and post‐exercise. Values are displayed as means and individual data points. ^‡^denotes a significant interaction effect (^‡^ *p* < 0.05/^‡‡^ *p* < 0.01), and ^#^denotes a significant effect of time point (^#^ *p* < 0.05/^##^ *p* < 0.01). Bars represent supplementation timing main effects. *N* = 20.

There was no significant difference between supplementation conditions in baseline plasma concentrations of TNF*α* ([TNF*α*]) (*p* = 0.114). [TNF*α*] was significantly elevated from baseline following exercise, irrespective of the supplementation condition (*p* = 0.002). There was a significant time and interaction effect on [TNF*α*] (Figure 4A). Analysis by training status showed a significant time and interaction effect on [TNF*α*] in the RA group (Figure 4B) but not the trained group (Figure 4C).

### 3.5 Plasma nitrate and nitrite concentrations

**FIGURE 5:**
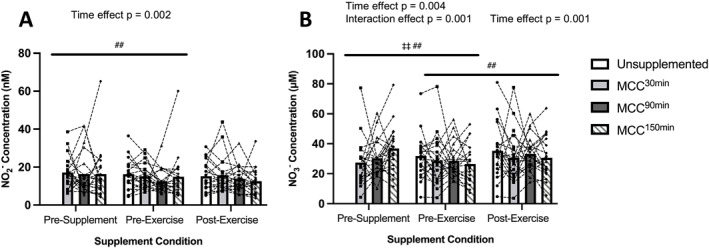
Comparison of unsupplemented control and Montmorency cherry concentrate (MCC) supplementation 30 min, 90 and 150 min prior to exercise for (A) plasma nitrite (NO_2_^−^) concentration for all participants (*N* = 20), (B) plasma nitrate (NO_3_^−^) concentration for all participants (*N* = 20), pre‐supplement, post‐supplement and post‐exercise. Values are displayed as means and individual data points. ^‡^denotes a significant interaction effect (^‡^ *p* < 0.05/^‡‡^ *p* < 0.01), and ^#^denotes a significant effect of time point (^#^ *p* < 0.05/^##^ *p* < 0.01). Bars represent supplementation timing main effects. *N* = 20.

[NO_2_^−^] decreased from pre‐supplementation to pre‐exercise for the whole cohort (*p* = 0.002 Figure 5A) and for the RA but not the trained group (SDC Figure S4A,B). There were no other significant time or supplement effects for [NO_2_^−^].

[NO_3_^−^] significantly decreased pre‐supplementation to pre‐exercise (time effect *p* = 0.004, interaction effect *p* = 0.001) and significantly increased pre‐to post‐exercise (Figure 5B SDC Figure S4C,D), time effect *p* = 0.001). There were no significant effects of supplementation on [NO_3_^−^]. There was no significant difference between conditions pre‐supplementation (*p* = 0.323).

### 3.6 Plasma phenolic metabolite concentrations

**FIGURE 6:**
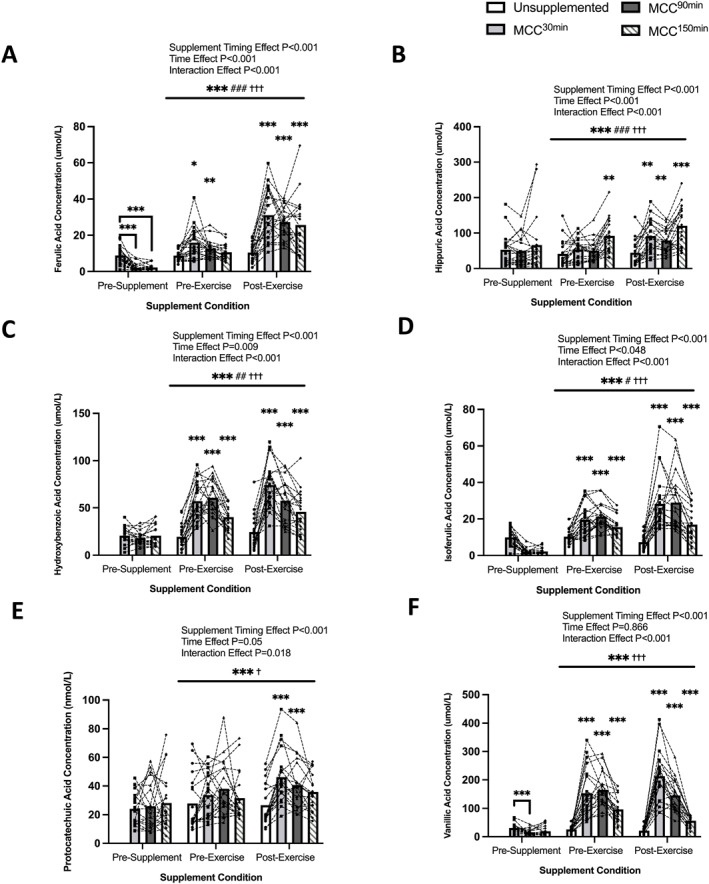
Comparison of unsupplemented control and Montmorency cherry concentrate (MCC) supplementation 30 min, 90 and 150 min prior to exercise for plasma concentrations of (A) ferulic acid (*N* = 20), (B) hippuric acid (*N* = 20), (C) hydroxybenzoic acid (HBA) (*N* = 20), (D) protocatechuic acid (PCA) (*N* = 20), (E) isoferulic acid (*N* = 20), (F) vanillic acid (*N* = 20), in all participants pre‐supplement, post‐supplement and post‐exercise. Values are displayed as means and individual data points. *denotes a significant effect of supplement timing (*p < 0.05/**p < 0.01/***p < 0.001), ^‡^denotes a significant interaction effect (^‡^ *p* < 0.05/^‡‡^ *p* < 0.01/^‡‡‡^ *p* < 0.001) and ^#^denotes a significant effect of time point (^#^ *p* < 0.05/^##^ *p* < 0.01/^###^ *p* < 0.001). Bars represent supplementation timing main effects. *N* = 20.

Plasma phenolic metabolites were significantly elevated after MCC supplementation (Figure 6). There were significant supplement condition, time and interaction effects for ferulic and hippuric acid (supplement effect: *p* < 0.001, time effect *p* < 0.001, interaction effect: *p* < 0.001), hydroxybenzoic acid (HBA) (supplement effect: *p* < 0.001, time effect: *p* = 0.009, interaction effect: *p* < 0.001), and isoferulic acid (supplement effect: *p* < 0.001, time effect: *p* = 0.048, interaction effect: *p* < 0.001). There were significant supplement and interaction effects for vanillic acid (supplement effect: *p* < 0.001, interaction effect: *p* = 0.018) and protocatechuic acid (PCA) (supplement effect: *p* < 0.001, interaction effect: *p* < 0.001). Total measured plasma phenolic metabolites showed significant supplement, time and interaction effects (supplement effect: *p* < 0.001, time effect: *p* < 0.001, interaction effect: *p* < 0.001) (Figure 6G).

There were no significant differences in pre‐supplementation plasma hippuric acid, HBA, PCA or isoferulic acid between conditions. However, plasma ferulic acid and vanillic acid concentrations were significantly higher pre‐supplementation for MCC^30min^ versus MCC^90min^ (*p* < 0.001) and MCC^30min^ versus MCC^150min^ for plasma ferulic acid (*p* < 0.001).

Plasma concentrations of ferulic acid, HBA, isoferulic acid and vanillic acid pre‐exercise were significantly higher for MCC^30min^ and MCC^90min^ versus US pre‐exercise (*p* < 0.01). For MCC^150min^ pre‐exercise plasma concentrations of hippuric acid, HBA, isoferulic acid and vanillic acid were also significantly greater than US (*p* < 0.01). Post‐hoc testing revealed significantly higher plasma concentrations of ferulic acid, hippuric acid, HBA, PCA, isoferulic acid and vanillic acid post‐exercise for MCC^30min^ and MCC^90min^ versus US pre‐exercise (*p* < 0.01). Plasma concentrations of ferulic acid, hippuric acid, HBA, isoferulic acid and vanillic acid were also greater post‐exercise for MCC^150min^ versus US (*p* < 0.01).

## 4 DISCUSSION

This study showed that acute supplementation with a single dose of MCC providing >800 mg polyphenols improved 15‐km cycling TT performance by 2.83% or 48.3 s, with the greatest effect over the first 5 km. This magnitude of improvement is comparable to that induced by caffeine supplementation, one of the most widely used ergogenic aids. Caffeine has consistently been shown to improve endurance performance by 2%–4% (Guest et al., 2021), albeit via different mechanisms of action. Supplementation 90‐min pre‐exercise produced the largest increase in plasma phenolic metabolites and improvement in performance, suggesting ergogenic effects are mediated by these metabolites. Trained but not RA participants experienced ergogenic effects from MC supplementation throughout the TT.

The results of the current study agree with those of a recent systematic review (Gao et al., 2020), which showed an overall significant improvement in endurance performance after polyphenol supplementation (∼11.5%, standardised mean difference: 0.36; 95% CI: 0.07, 0.64; *p* = 0.01). Following supplementation, plasma phenolic metabolites were elevated pre‐ and post‐exercise relative to the pre‐supplementation baseline, irrespective of supplementation timing. Concentrations were greatest pre‐exercise in the MCC^90min^ condition, where the greatest improvements in TT performance were also observed. Further, concentrations of measured plasma phenolic metabolites were greatest post‐exercise in the MCC^30min^ condition. Given this timepoint represents a period ∼75–90 min post‐supplementation (depending on TT^Time^), these findings further support previous literature showing that plasma phenolic metabolites peak 1–2 h following polyphenol ingestion (Keane, Bell, et al., 2016) and suggest that phenolic metabolites are likely to be key mediators of the observed MC performance effects. However, it should be noted that plasma values only provide a proxy for intra‐muscular uptake of phenolic metabolites, and there is currently a lack of research investigating the muscle uptake of polyphenols (Stromsnes et al., 2021).

The prevailing hypothesised mechanism underpinning improvements in endurance performance from MC supplementation is an increase in the bioavailability of the potent vasodilator NO (Cook et al., 2017; Senefeld et al., 2020; Trexler et al., 2014) and potentially increased muscle perfusion. All phenolic metabolites measured in the current study have been shown to significantly correlate with FMD measured following both acute (2 h) and chronic (28 days) supplementation (Rodriguez‐Mateos et al., 2019). Vanillic (*r* = 0.60 Pearson's correlation) and isoferulic acid (*r* = 0.43) have the strongest correlation with FMD (Rodriguez‐Mateos et al., 2019). Furthermore, improvements observed previously in FMD have been shown to occur in conjunction with reductions in the activity of NADPH oxidase coinciding with a peak in plasma phenolics 1–2 h post‐ingestion (Rodriguez‐Mateos et al., 2013). This may support the hypothesis that NO bioavailability is enhanced through phenolic acid mediated inhibition of superoxide producing enzymes (Maraldi, 2013) resulting in reduced loss of NO via conversion to peroxynitrite.

However, similar to previous studies (Davis et al., 2017; Keane et al., 2016b, 2018), we found no significant effect of supplementation on plasma [NO_3_^−^] or [NO_2_^−^] before, during or after exercise. Although not during exercise, changes in [NO_2_^−^] and [NO_3_^−^] have been observed following MC supplementation, during recovery from temporary ischemia‐reperfusion injury induced by prolonged forearm occlusion (Aboo Bakkar et al., 2019). However, [NO_3_^−^] and [NO_2_^−^] are relatively poor proxy measures and may not indicate NO bioavailability.

Whilst there is limited evidence for improved NO bioavailability in the present study, previous research has demonstrated increased muscle oxygenation at rest following MC supplementation (Morgan et al., 2019). However, similar to the current study and others in this area (Davis et al., 2017; Keane et al., 2018; Morgan et al., 2019), this effect was not maintained into exercise. This may be due to a lack of sensitivity in the NIRS method, especially during exercise which introduces confounding factors such as increased skin blood flow in response to increased core body temperature (Davis et al., 2006). NIRS values do not always reflect venous oxygen saturation during SSE, with an association between muscle oxygenation and femoral vein O_2_ saturation observed at exercise onset but not persisting following the attainment of a steady‐state plateau (Costes et al., 1996; MacDonald et al., 1999).

Another potential mechanism for performance improvements observed is an analgesic or anti‐hyperalgesic effect. In the current study, higher TT capillary [lactate] and HR were observed following MC supplementation, which may suggest an enhanced ability to tolerate higher levels of pain induced by factors like muscle acidosis or perhaps an increase in central drive. MC anthocyanins have been shown to reduce pain in animal models (Tall et al., 2004), an effect similarly observed in humans during endurance exercise (Kuehl et al., 2010).

Mechanisms behind the potential analgesic or anti‐hyperalgesic effect of MC supplementation may be related to anti‐inflammatory effects of polyphenols. MC has been suggested to induce a similar anti‐inflammatory response to pharmacological analgesics (13) widely used by athletes (Lundberg et al., 2018) which can also enhance performance (Holgado et al., 2018; Seeram et al., 2001). Indeed, ingestion of 1.5 g of paracetamol has been shown to increase trained cyclists' power output and improve 16.1‐km cycling TT performance (26:15 ± 1:36 min vs. placebo: 26:45 ± 2:02 min) (Mauger et al., 2010) by a similar magnitude to TT^Time^ improvement in the current study. In the current study, [TNF*α*] increased post‐exercise for all conditions, except for MCC^150min^, which, whilst faster than US, was the slowest of the supplement conditions. This may suggest that performance enhancement is not related to anti‐inflammatory effects; however, [TNF*α*] is only a proxy measure of intra‐muscular concentrations (Jajtner et al., 2016) and the inflammatory response in muscle. Furthermore, there is likely to be a time delay in observable circulatory increases compared to intra‐muscular increases, which would also impact how the appearance of circulatory markers coincides with supplementation timing.

Reduced ROS production, via inhibition of superoxide‐producing enzymes such as NADPH oxidase, may contribute to the ergogenic effects of MC by preserving redox‐sensitive processes like afferent nerve transmission and sarcoplasmic reticulum calcium handling (Gandevia, 2001; Powers et al., 2008). Previous literature has shown that polyphenol supplementation can attenuate concentrations of plasma F2‐isoprostanes during running at 72% and 60% of *V̇*O_2max_ (Davison et al., 2012; McAnulty et al., 2011). Furthermore, both chronic (7 days Chang et al., 2010 and 3 weeks Fuster‐Munoz et al., 2016) and acute polyphenol supplementation (15‐min pre‐exercise and at 15‐min intervals during exercise (Morillas‐Ruiz et al., 2006) have been shown to reduce markers of OS following exercise.

The primary mechanism for improved TT performance following MCC supplementation observed in the present study remains unclear. However, enhanced performance occurs in parallel with elevated plasma phenolic acids and likely occurs through a combination of the aforementioned mechanisms: improved NO bioavailability; anti‐hyperalgesic effects via anti‐inflammatory effects; and reduced effects of ROS via the inhibition of ROS producing enzymes.

Analysis by training status showed a significant improvement in TT^Time^ for the trained but not the RA group. Similarly, analysis by distance split showed TT^Time^ performance was significantly improved at TT^5km^ for both trained and RA participants, but the advantage gained within the first 5 km was only maintained by the trained population. These results are in contrast with previous evidence from the literature, where studies employing NZB extract or pomegranate supplementation have observed performance benefits for both RA and trained individuals, but with the size of the effect generally greater in RA individuals.

Whilst these previous studies supplemented participants for 7‐day pre‐exercise performance, and thus are not directly comparable to the current study, final doses were provided at 30 min (Trinity et al., 2014) and 2 h prior to exercise, respectively (Cook et al., 2015; Murphy et al., 2017). Interestingly, in line with our findings, only studies supplementing 2‐h pre‐exercise demonstrated enhanced performance (for 16.1 and 4 km cycling TTs).

Seven days of powdered NZB extract supplementation (105 mg of polyphenols) improved cycling TT performance over 16.1 km in RA individuals (2.4% compared to 3.7% in the current study) (Cook et al., 2015). However, in trained individuals, NZB extract supplementation only yielded a small improvement of 0.8% (compared to 1.9% in the current study) for repeated cycling TT performance, though this was over a shorter distance of 4 km (Murphy et al., 2017). Furthermore, supplementation for 7 days with pomegranate (∼1800 mg of polyphenols) produced no significant improvement in 10‐min cycling TT performance in trained participants (Trinity et al., 2014). However, it could be suggested that power output over 10 min is less ecologically valid than a distance TT. In the current study, whilst a significant improvement in TT^Time^ was only found for trained participants, improvement in TT^Time^ was 3.7 ± 5.5% for the RA group compared to 1.9 ± 1.9% in the trained group. These results suggest there may have been a lack of statistical power in the RA group, probably due to a greater intra‐ and inter‐individual variation in TT^Time^.

The current study used an unsupplemented rather than a placebo control condition. Whilst this lack of blinding for the unsupplemented condition represents an important limitation, a crossover design including a placebo condition at each supplementation timepoint would have required participants to complete 6 conditions. This would represent an unacceptably high participant burden and further lengthen the study duration introducing variations in participant background status. A crossover design is preferable to alternatives, particularly considering the large intra‐ and inter‐individual variation in TTTime within the RA group. Whilst the absence of a placebo should be noted when considering our results, so too should the presence of three active conditions to which participants were blind. It is notable that in the context of careful counterbalancing of ‘active’ conditions, a significant performance benefit was demonstrated only at the supplementation timepoint coinciding with a biochemical outcome measure (peak plasma phenolic concentrations) unlikely to have been influenced by participant expectation.

## 5 CONCLUSION

Acute MC supplementation enhanced 15‐km cycling TT performance, with optimal timing for supplementation 90 min prior to exercise, maximising phenolic metabolite exposure during exercise. Our findings suggest supplementation is effective for trained but not RA individuals. However, more research is required to mitigate for the influence of variability in performance on the effects in RA individuals. Mechanisms of action remain unclear but seem to be driven by the elevation of phenolic metabolites and may involve analgesic effects alongside reduced superoxide production and enhanced NO bioavailability with the resulting enhancement of vascular and contractile functions.

## CONFLICT OF INTEREST STATEMENT

The authors have no competing interests/conflicts of interest to disclose.

## PARTICIPANT CONSENT STATEMENT

All participants to this study provided written, informed consent prior to data collection.

## Supporting information

Supporting Information S1

## Data Availability

The data that supports the findings of this study are available in the supplementary material of this article.
