# Supplementary material for: Montmorency cherry supplementation enhances 15 km cycling time trial performance: Optimal timing 90‐min pre‐exercise
Source: Eur J Sport Sci. 2024 Aug 30;24(10):1480–94. doi: 10.1002/ejsc.12187 (PMC11451560; doi:10.1002/ejsc.12187)
Supplement: Supplementary file 1 — Supporting Information S1 [file EJSC-24-1480-s001.pdf]

Supplementary Digital Content

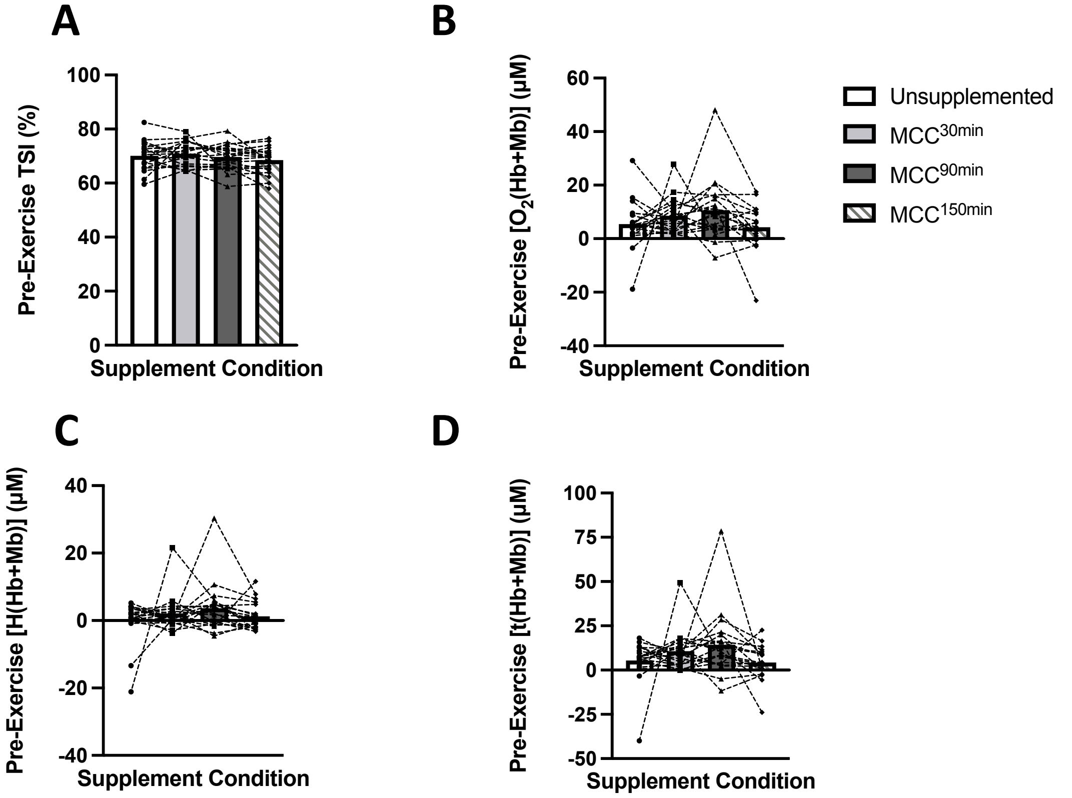

Figure 1: Effect of unsupplemented control and Montmorency Cherry Concentrate (MCC) supplementation 30 minutes, 90 minutes and 150 minutes prior to exercise of **A:** mean tissue saturation index (TSI) **B:** mean combined oxyhaemoglobin and myoglobin [O_2(Hb+Mb)] **C:** mean deoxyhaemoglobin and myoglobin [H(Hb+Mb)] **D:** mean combined (total) oxy and deoxyhaemoglobin and myoglobin [t(Hb+Mb)]over 20 seconds pre-exercise. Values are displayed as means and individual data points. N = 20.

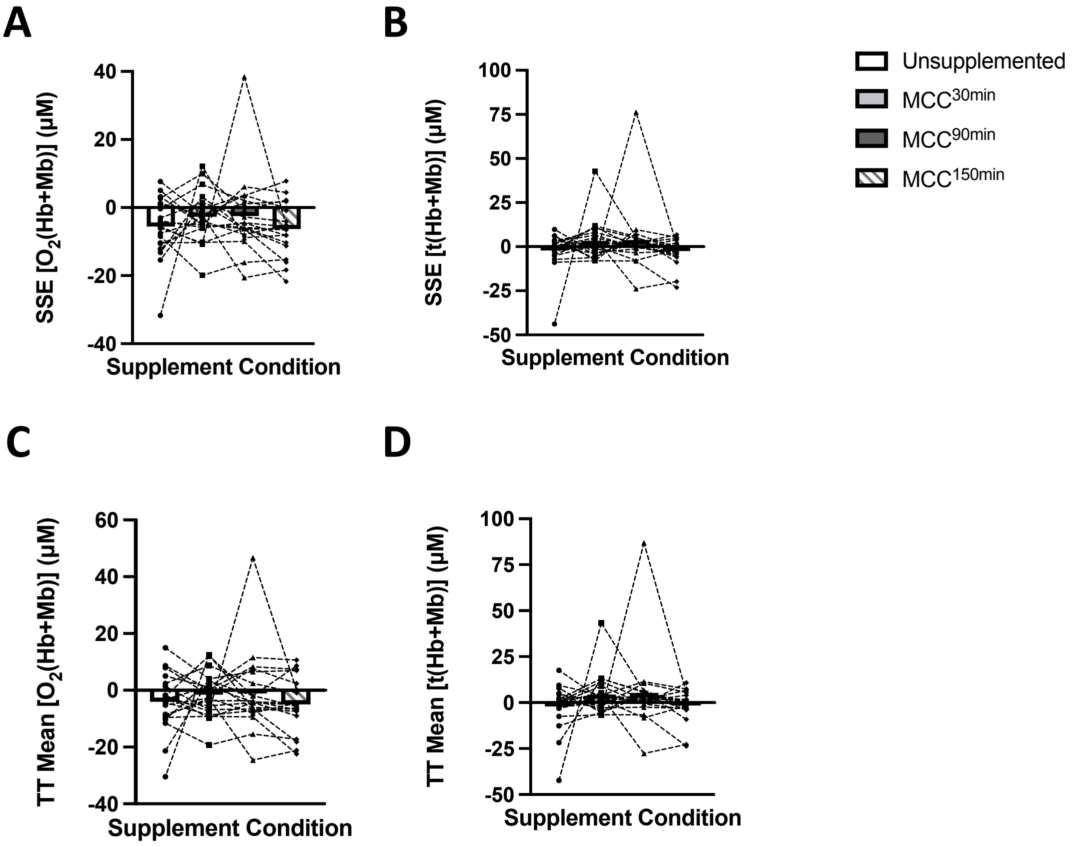

Figure 2: Effect of unsupplemented control and Montmorency Cherry Concentrate (MCC) supplementation 30 minutes, 90 minutes and 150 minutes prior to exercise on **A:** mean combined oxyhaemoglobin and myoglobin [O_2(Hb+Mb)] during steady state exercise (SSE) **B:** mean combined (total) oxy and deoxyhaemoglobin and myoglobin [t(Hb+Mb)] during SSE **C:** mean combined oxyhaemoglobin and myoglobin [O_2(Hb+Mb)] during time trial (TT) **D:** mean combined (total) oxy and deoxyhaemoglobin and myoglobin [t(Hb+Mb)] during TT. Values are displayed as means and individual data points. N = 20.

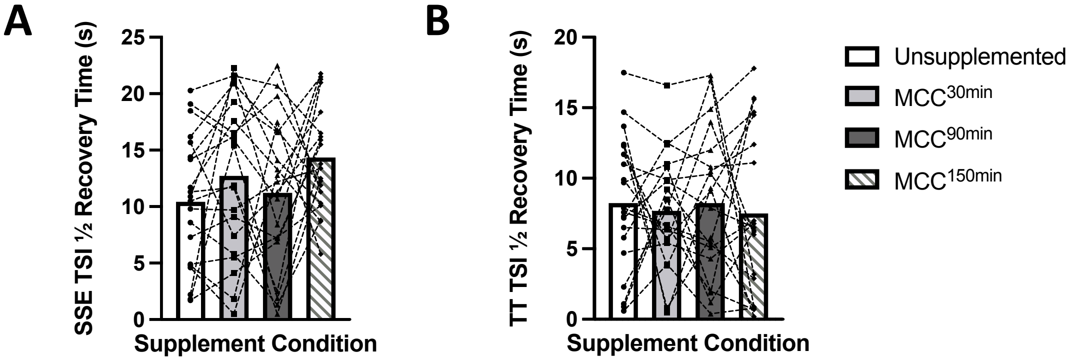

Figure 3: Effect of unsupplemented control and Montmorency Cherry Concentrate (MCC) supplementation 30 minutes, 90 minutes and 150 minutes prior to exercise of **A:** tissue saturation index (TSI) half recovery time following steady state exercise (SSE) **B:** TSI half recovery time following time trial (TT). Values are displayed as means and individual data points. N = 20.

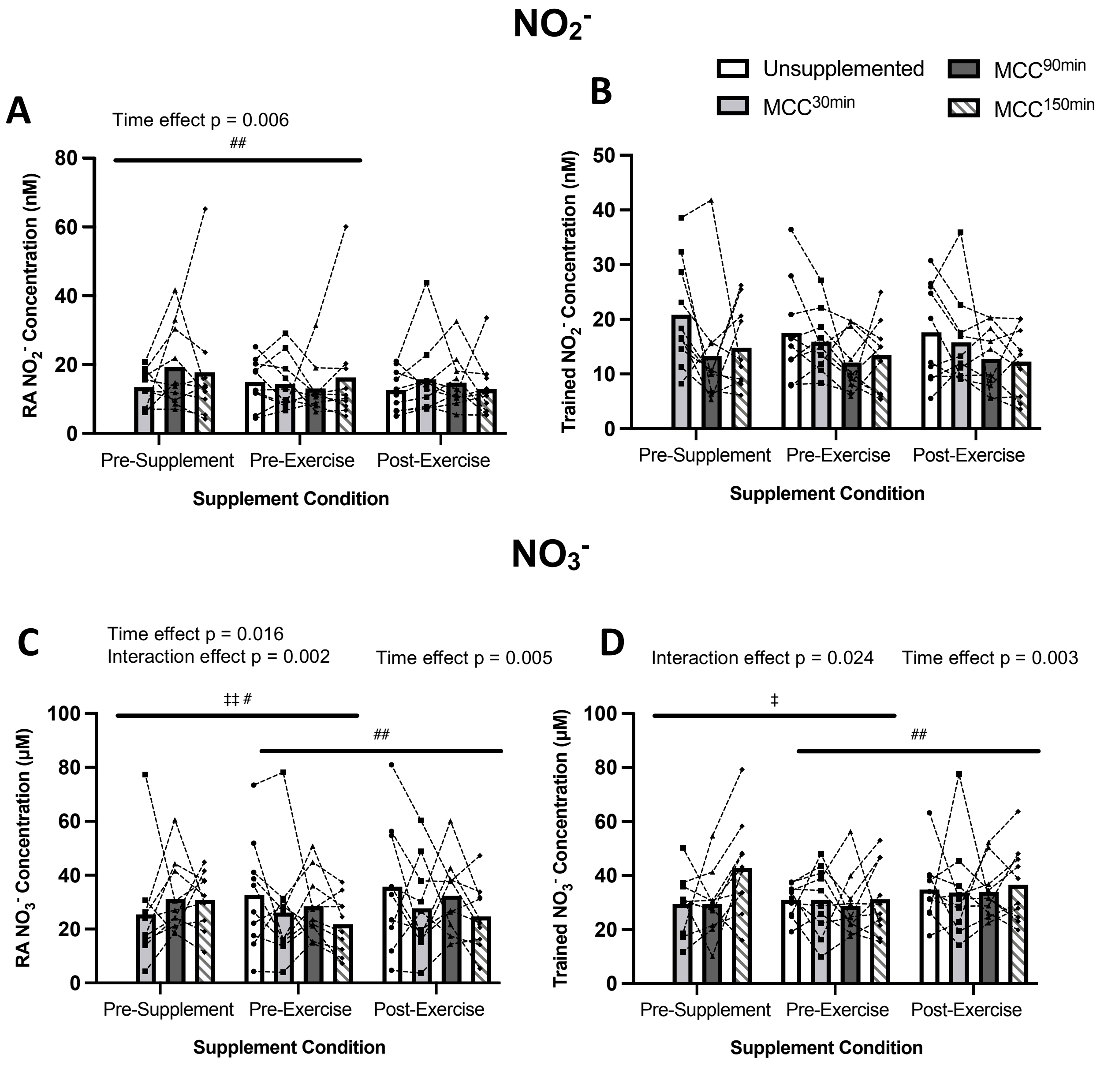

Figure 4: Comparison of unsupplemented control and Montmorency Cherry Concentrate (MCC) supplementation 30 minutes, 90 minutes and 150 minutes prior to exercise for **A:** plasma nitrite (NO_2^-) concentration for for recreationally active participants (N = 10) **B:** plasma nitrate (NO_2^-) for trained participants (N = 10) **C:** plasma nitrate (NO_3^-) for recreationally active participants (N = 10) **D:** plasma nitrate (NO_3^-) for trained participants (N = 10), pre-supplement, post-supplement and post-exercise. Values are displayed as means and individual data points. ‡denotes a significant interaction effect (‡$p<0.05$/‡‡$p<0.01$), #denotes a significant effect of time point (#$p<0.05$/##$p<0.01$). Bars represent supplementation timing main effects. N = 20.

Table 1: Performance level parameters (adapted from De Pauw et al., 2013 and Decroix et al., 2016).

Performance Level	$\dot{V}O_{2max}$ (mL.kg^{-1}.min^{-1})		Peak Power Output (W.kg^{-1})	
	Males	Females	Males	Females
1	<45.0	<37.0	>4.0	<3.0
2	45.0-54.9	37.0-48.0	3.6-4.5	3.0-3.8
3	55.0-64.9	48.0-52.0	4.6-5.5	3.8-4.3
4	65.0-71.0	52.0-58.0	4.9-6.4	4.3-5
5	>71.0	>58	>5.5	>5

Table 2: Phenolic content of MCC supplement

Analyte	Result	Unit
Malvidin	6.757	mg / ml
Cyanidin	0.242	mg / ml
Pelargonidin	22.65	mg / ml
Peonidin	0.803	mg / ml
Delphinidin	11.28	mg / ml
Petunidin	0.901	mg / ml
Total Anthocyanindins	42.631	mg / ml
Total Anthocyanins (calc. as mono-glycosides)	51.98	mg / ml
Melatonin	2.615	mcg / ml
Polyphenolics	13.86	mg/ ml

Table 3: Standardised breakfast nutritional information (1 46 g sachet)

Nutritional Breakdown	Content
Calories	179 Kcal
Energy	750 kJ
Protein	4.6 g
Total Fat	3.5 g
- Saturated Fat	0.8 g
Carbohydrate	30.5 g
- Sugars	8.9 g
Dietary Fibre	3 g
Sodium	10 mg

Table 4: Phenolic metabolite assay characteristics

	Protocatechuic acid<br>nmol/L	4-hydroxybenzoic acid<br>nmol/L	hippuric acid<br>µmol/L	vanillic acid<br>nmol/L	ferulic acid<br>nmol/L	isoferulic acid<br>nmol/L
Linearity	0.5-486	1.0-749	0.01-800	0.5-336.7	0.5-321.1	0.5-337.7
Typical r^2	>0.98	>0.98	>0.98	>0.98	>0.98	>0.98
Intra assay imprecision conc. mean (%CV), n=6	39.4 (2.6)	57.3 (3.5)	6.1 (0.3)	36.9 (9.5)	3.3 (6.6)	2.1 (4.0)
	79.4 (5.8)	261.6 (2.5)	34.7 (0.9)	43.7 (5.9)	9.4 (3.7)	106.2 (4.2)
	430.4 (2.2)	622.9 (2.0)	499.2 (2.4)	250.9 (7.6)	143.9 (2.1)	218.6 (4.4)
Inter assay imprecision conc. mean (%CV), n=6	20.0 (10.6)	32.9 (6.0)	32.9 (6.0)	35.7 (5.4)	4.5 (6.1)	3.7 (9.9)
	135.1 (9.0)	393.6 (6.2)	393.6 (6.2)	221.6 (6.8)	132.2 (6.1)	133.6 (6.5)
	424.7 (7.0)	622.8 (7.6)	522.8 (7.6)	359.4 (9.2)	294 (7.0)	277 (7.5)
Lower Limits of quantification (LLoQ)	1.0	1.0	0.05	1.0	1.0	1.0
Spiked recovery Mean% (±SD)*	98.5% (±2)	96.8% (±3)	103% (±2)	105% (±2)	99% (±2)	102% (±3)

* Base serum used for spiking contained 100 µmol.L^{-1} of hippuric acid and no other endogenous phenolic metabolites. Each spiked sample was tested six times.

Table 5: Physiological and perceived exertion metrics collected during steady state exercise for an unsupplemented condition and Montmorency cherry concentration supplementation 30, 90 and 150 minutes prior to exercise. Data is displayed for recreationally active participants (N=10), trained participants (N=10) and all participants (total, N=20). Main effect of supplement condition is displayed for all three groups.

	Unsupplemented			MCC^{30mins}			MCC^{90mins}			$MCC^{150mins}$			Supplement Effect (*p*)		
	RA	Trained	Total	RA	Trained	Total	RA	Trained	Total	RA	Trained	Total	RA	Trained	Total
Exercise Economy ($L.min^{-1}$)	2.70 ± 0.69	3.29 ± 0.86	3.00 ± 0.81	2.76 ± 0.68	3.36 ± 0.76	3.06 ± 0.77	2.79 ± 0.51	3.19 ± 0.70	2.99 ± 0.63	2.76 ± 0.60	3.21 ± 0.69	2.99 ± 0.67	0.701	0.116	0.454
Average HR (BPM)	149 ± 9	162 ± 8	156 ± 11	150 ± 15	162 ± 9	156 ± 14	148 ± 11	162 ± 13	155 ± 14	150 ± 8	162 ± 10	156 ± 11	0.972	0.998	0.986
Capillary [Lactate] Δ ($mmol.L^{-1}$)	6.1 ± 2.4	8.9 ± 4.5	7.5 ± 3.8	5.9 ± 2.2	7.9 ± 2.6	6.9 ± 2.6	6.3 ± 3.4	8.3 ± 4.8	7.3 ± 4.2	6.6 ± 2.6	7.0 ± 3.8	6.8 ± 3.2	0.863	0.219	0.662
End Exercise RPE	14.2 ± 1.9	15.8 ± 1.9	15.0 ± 2.0	14.3 ± 2.3	15.7 ± 1.4	15.0 ± 2.0	13.8 ± 1.5	15.2 ± 1.5	14.5 ± 1.6	14.8 ± 2.5	15.0 ± 1.3	14.9 ± 1.9	0.207	0.052	0.168

Montmorency cherry concentration supplementation 30, 90 and 150 prior to exercise (MCC^{30mins}/MCC^{90mins}/$MCC^{150mins}$), recreationally active (RA), Beats per minute (BPM), rate of perceived exertion (RPE).

Table 6: Physiological and perceived exertion metrics collected during time trial exercise for an unsupplemented condition and Montmorency cherry concentration supplementation 30, 90 and 150 minutes prior to exercise. Data is displayed for recreationally active participants (N=10), trained participants (N=10) and all participants (total, N=20) by 5km split. Main effect of supplement condition is displayed for all three groups.

		US			MCC30mins			MCC90mins			MCC150mins			Supplement Effect (*p*)		
		RA	Trained	Total	RA	Trained	Total	RA	Trained	Total	RA	Trained	Total	RA	Trained	Total
Pre-TT	HR (BPM)	106 ± 15	106 ± 15	106 ± 15	105 ± 15	108 ± 12	107 ± 13	106 ± 13	108 ± 13	107 ± 12	107 ± 20	105 ± 10	106 ± 16	0.734	0.796	0.738
	Capillary [Lactate] ($mmol.L^{-1}$)	7.0 ± 2.9	7.4 ± 3.9	7.2 ± 3.3	7.6 ± 2.6	7.4 ± 3.6	7.5 ± 3.1	7.8 ± 3.1	7.2 ± 4.3	7.5 ± 3.7	7.6 ± 3.3	6.2 ± 2.9	6.9 ± 3.1	0.899	0.414	0.800
	Borg RPE	10.0 ± 1.8	9.4 ± 2.0	9.7 ± 1.8	9.7 ± 2.5	9.6 ± 2.3	9.7 ± 2.3	9.2 ± 2.2	9.6 ± 1.7	9.4 ± 1.9	10.0 ± 1.3	9.1 ± 2.0	9.6 ± 1.7	0.321	0.641	0.807
5km Split	HR (BPM)	160 ± 18	170 ± 6	165 ± 14	163 ± 16	174 ± 5	168 ± 13*	164 ± 19	176 ± 8	170 ± 15*	165 ± 13	173 ± 6	169 ± 11	0.212	0.036*	0.009**
	Capillary [Lactate] ($mmol.L^{-1}$)	7.7 ± 4.1	9.9 ± 4.7	8.8 ± 4.5	9.3 ± 4.1	10.8 ± 3.3	10.0 ± 3.7	10.1 ± 4.6	11.2 ± 4.1	10.7 ± 4.3	9.4 ± 3.4	10.4 ± 2.8	9.9 ± 3.1	0.248	0.604	0.149
	Borg RPE	14.4 ± 2.0	15.3 ± 1.2	14.9 ± 1.6	14.5 ± 2.4	15.8 ± 1.5	15.2 ± 2.0	14.4 ± 3.2	15.3 ± 1.7	14.9 ± 2.5	14.3 ± 1.8	15.4 ± 1.3	14.9 ± 1.6	0.991	0.449	0.782

10km Split	HR (BPM)	162 ± 20	173 ± 6	167 ± 15	166 ± 16	177 ± 4	171 ± 12	166 ± 17	177 ± 6	172 ± 14	170 ± 20	175 ± 5	173 ± 15	0.066	0.080	0.011*
	Capillary [Lactate] ($mmol.L^{-1}$)	7.5 ± 4.1	10.3 ± 3.1	8.9 ± 3.8	9.2 ± 4.5	11.9 ± 4.1	10.6 ± 4.4**	10.3 ± 4.3**	12.2 ± 4.2	11.2 ± 4.2**	9.7 ± 4.1	10.7 ± 3.3	10.2 ± 3.7	0.002**	0.171	0.001***
	Borg RPE	15.6 ± 1.8	17.1 ± 1.5	16.4 ± 1.8	15.6 ± 2.9	17.5 ± 1.4	16.6 ± 2.4	15.8 ± 3.5	17.2 ± 1.4	16.5 ± 2.7	15.8 ± 2.4	17.2 ± 1.0	16.5 ± 1.9	0.977	0.661	0.950
15km Split	HR (BPM)	180 ± 14	184 ± 8	182 ± 11	183 ± 14	186 ± 6	184 ± 10	181 ± 19	186 ± 7	184 ± 14	182 ± 15	186 ± 7	184 ± 12	0.485	0.607	0.822
	Capillary [Lactate] ($mmol.L^{-1}$)	11.3 ± 2.6	13.6 ± 3.0	12.4 ± 3.0	12.6 ± 3.9	14.8 ± 2.5	13.7 ± 3.4	14.5 ± 3.3	13.9 ± 4.1	14.2 ± 3.6	13.1 ± 4.0	14.1 ± 2.5	13.6 ± 3.3	0.182	0.625	0.323
	Borg RPE	17.9 ± 2.6	18.9 ± 1.0	18.4 ± 2.0	17.2 ± 2.7	19.7 ± 0.5	18.5 ± 2.3	17.5 ± 2.7	19.5 ± 0.7	18.5 ± 2.2	17.4 ± 2.7	19.7 ± 0.7	18.6 ± 2.2	0.337	0.012*	0.925

Montmorency cherry concentration supplementation 30, 90 and 150 prior to exercise (MCC^{30mins}/MCC^{90mins}/$MCC^{150mins}$), recreationally active (RA), Beats per minute (BPM), rate of perceived exertion (RPE).
